# EFHD2 promotes epithelial-to-mesenchymal transition and correlates with postsurgical recurrence of stage I lung adenocarcinoma

**DOI:** 10.1038/s41598-017-15186-y

**Published:** 2017-11-03

**Authors:** Chi-Chen Fan, Wei-Chung Cheng, Yu-Chuen Huang, Yuh-Pyng Sher, Nia-Jhen Liou, Yu-Chuan Chien, Pei-Shan Lin, Pei-Syuan Lin, Chung-Hsuan Chen, Wei-Chao Chang

**Affiliations:** 1 Department of Superintendent Office, Mackay Memorial Hospital, Taipei, Taiwan; 2 Department of Medical Laboratory Science and Biotechnology, Yuanpei University, Hsinchu, Taiwan; 3 Research Center for Tumor Medical Science, China Medical University, Taichung, Taiwan; 4 Graduate Institute of Biomedical Sciences, China Medical University, Taichung, Taiwan; 5 School of Chinese Medicine, China Medical University, Taichung, Taiwan; 6 Genetics Center, Department of Medical Research, China Medical University Hospital, Taichung, Taiwan; 7 Center for Molecular Medicine, China Medical University Hospital, Taichung, Taiwan; 8 Genomics Research Center, Academia Sinica, Taipei, Taiwan; 9 Institute of Biochemistry & Molecular Biology, National Yang-Ming University, Taipei, Taiwan; 10 Department of Chemistry, National Taiwan University, Taipei, Taiwan; 11 Institute of Atomic & Molecular Sciences, Academia Sinica, Taipei, Taiwan

## Abstract

Surgery is the only curative treatment for early-stage non-small cell lung cancer (NSCLC) patients. However, approximately one-third of these patients develop recurrence, which remains the main cause of mortality in the postsurgical treatment of NSCLC. Many molecular markers have been proposed to predict recurrence of early-stage disease, but no marker has demonstrated sufficient reliability for clinical application. In the present study, the novel protein EF-hand domain-containing protein D2 (EFHD2) was identified as expressed in highly metastatic tumor cells. EFHD2 increased the formation of protrusive invadopodia structures and cell migration and invasion abilities and promoted the epithelial-to-mesenchymal transition (EMT) character of lung adenocarcinoma cells. We demonstrated that the mechanism of EFHD2 in enhancing EMT occurs partly through inhibition of caveolin-1 (CAV1) for cancer progression. The expression of EFHD2 was significantly correlated with postsurgical recurrence of patients with stage I lung adenocarcinoma in the Kaplan-Meier-plotter cancer database search and our retrospective cohort study (HR, 6.14; 95% CI, 2.40–15.74; *P* < 0.001). Multivariate Cox regression analysis revealed that EFHD2 expression was an independent clinical predictor for this disease. We conclude that EFHD2 expression is associated with increased metastasis and EMT and could serve as an independent marker to predict postsurgical recurrence of patients with stage I lung adenocarcinoma.

## Introduction

Non-small cell lung cancer (NSCLC) is one of the most common causes of cancer death in the developed world^1^. Surgical resection is the standard treatment for patients with early-stage NSCLC. However, the relatively high risk of recurrence remains the main cause of mortality in the postsurgical treatment of NSCLC. According to clinical statistics, 30–35% stage I NSCLC patients subjected to surgical treatment experience local and/or distant recurrence during a 5-year follow-up period^2,3^. The 5-year survival rate is 66–84% in stage IA and 53–66% in stage IB^4^.

In recent decades, considerable effort has been made to identify suitable factors related to postsurgical recurrence using clinical parameter or molecular marker approaches^5,6^. For precise prediction of tumor recurrence, proteins could serve as a more ideal marker to monitor cancer prognosis because proteins are highly relevant to biological functions. Thus, most currently available markers are proteins. Numerous strategies have been used to discover markers for recurrence prediction, including comparative analysis by comparing the differential protein signature between patients’ tissues with and without recurrence or by comparing the protein changes between primary and metastatic tissues in the same patient. The combination of known malignant factors as an integrated marker set to predict cancer recurrence is another commonly used method. Given that no marker developed by traditional methods has been sufficiently effective for clinical application, we choose an alternate approach to uncover novel markers. Although tumors exhibit significant histological heterogeneity, we sought to assess whether tumor cells use a similar mechanism to promote metastasis at a very early stage of disease. Thus, we assumed that common proteins are involved in metastasis at an early stage of disease in various cancers. Based on this assumption, we combined comparative proteomics and intersectional analysis to identify metastasis-related proteins from three pairs of tumor cells. Surprisingly, only one candidate, namely, EF-hand domain-containing protein D2 (EFHD2), exhibited overexpression in all highly metastatic tumor cell lines.

EFHD2 is a 27-kDa highly conserved calcium-binding protein that is located in membrane lipid rafts^7^. EFHD2 is widely expressed in brain and immune cells, including B cells, CD4 + /CD8 + T cells, natural killer cells, and peripheral blood mononuclear cells^8^. EFHD2 is involved in immune cell activation through regulating the activity of Src kinase, a key factor for the signaling of adaptive and innate immune receptors^9^. EFHD2 dysfunction is associated with autoimmune and neuropathological diseases, such as rheumatoid arthritis^10^, Parkinson’s disease^11^, and Alzheimer’s disease^12^. EFHD2 is characterized as an actin-binding protein that induces the formation of actin bundling through its EF-hand motif and the coiled-coil domain^13^. The interaction between EFHD2 and F-actin modulates membrane dynamics, such as lamellipodia formation^14^. Recently, EFHD2 enhances cell migration velocity by activating the Rho family of small GTPases in mouse B16F10 melanoma cells, suggesting the potential role of EFHD2 in cancer metastasis^15^. In lung cancer, the correlation between EFHD2 and cancer metastasis is unclear, and whether EFHD2 is related to postsurgical recurrence of stage I NSCLC remains to be determined.

The scaffold protein caveolin-1 (CAV1), a principal component of caveolar membrane coat, regulates multiple cancer-associated processes, including cellular transformation, tumor growth and survival, cell migration and metastasis, and multidrug resistance^16^. CAV1 harbors tumor suppressor or oncoprotein properties dependent on disease stage^17^ and cancer type^18^. CAV1 inhibits the cell cycle and cell proliferation at the early stage of tumorigenesis, whereas it induces progressive processes, such as metastasis, at late stage of disease^19^. In lung cancer, reduced CAV1 protein levels are commonly observed^20^. CAV1 mRNA and protein levels were downregulated during cell transformation by oncogenes, such as Ha-Ras, v-Abl, Myc, and Neu, suggesting a negatively regulatory role of CAV1 in tumor development^21^. In addition, the suppression of CAV1 leads to the activation of endocytosis^22^, which is involved in the regulation of cell migration and cancer metastasis^23^. Through a comparative proteomic analysis, CAV1 was identified to exhibit negative regulation by EFHD2 in the present study. Thus, we explored the mechanism of EFHD2 in increasing the metastatic ability of lung tumor cells via suppression of CAV1.

## Materials and Methods

### Cell lines and cell culture

Human colorectal cancer cells SW480 and SW620, human bronchial epithelial cell line BEAS-2B, and lung adenocarcinoma cells A549 and H1299 were obtained from the American Type Culture Collection (ATCC, Maryland, USA). Human esophageal cancer cells CE81T/VGH and CE146T/VGH were obtained from the Bioresource Collection and Research Center (BCRC, Hsinchu, Taiwan). Human lung adenocarcinoma cell lines CL1-0 and CL1-5-F4 (F4) were a gift from Dr. Pan-Chyr Yang (National Taiwan University, Taipei, Taiwan), and CL1-5 was established by selection of increasingly invasive cell populations from CL1-0^24^. Bm7BrM was derived from F4 with specific brain metastasis^25^. BEAS-2B, H1299, and F4 were cultured in DMEM/F-12 media (Invitrogen). A549, CL1-0, and Bm7BrM were maintained in RPMI 1640 media (Invitrogen), and H2981 were maintained in DMEM media (Invitrogen). All culture media were supplemented with 10% fetal bovine serum and 1% antibiotics (GIBCO). All cells were grown in a humidified atmosphere of 5% CO_2_ and 95% air at 37 °C.

### Tissue specimens

This study protocol (CMUH103-REC1-140) was approved by the Regional Ethical Committee and the Institutional Review Board of China Medical University Hospital (Taichung, Taiwan) according to the guidelines of the Helsinki Convention. Tissue specimens without linkage of personal privacy were obtained from patients with stage I lung adenocarcinoma who underwent pulmonary resection at China Medical University Hospital from Jan 2008 to Mar 2010. The specimens from patients who received neoadjuvant chemotherapy were excluded. A total of 50 samples were divided into two groups based on postsurgical recurrence: (1) recurrence group: samples from patients suffering from recurrence within 5-year of surgery (*N* = 18), and (2) non-recurrence group: samples from patients without recurrence within 5-year of surgery (*N* = 32). Several demographic, clinical and biochemical variables, such as age, gender, pathologic stage, tumor location, differentiation, smoking status, EGFR mutation, and EFHD2 expression were evaluated for the consideration of recurrence (Table 1). For EGFR mutation determination, tumor DNA extracted from paraffin blocks was subjected to PCR amplification and sequence analysis of the EGFR gene at exons 18, 19, 20, 21, and 22^26^.

**Table 1:** Association between postsurgical recurrence and clinical and pathological characteristics in stage I lung adenocarcinoma patients.

Characteristics		Patients’ condition		*P*-value^a^
		Recurrence	Non-recurrence	
Age	<68	7 (31.8%)	15 (68.2%)	0.585
	≧68	11 (39.3%)	17 (60.7%)	
Gender	Man	10 (43.5%)	13 (56.5%)	0.309
	Female	8 (29.6%)	19 (70.4%)	
Smoking status	Ever	7 (35.0%)	13 (65.0%)	0.904
	Never	11 (36.7%)	19 (63.3%)	
Pathologic stage	IA	8 (24.2%)	25 (75.8%)	*0.016*
	IB	10 (58.8%)	7 (41.2%)	
Tumor location	LLL	3 (42.9%)	4 (57.1%)	0.965
	LUL	5 (35.7%)	9 (64.3%)	
	RLL	2 (25.0%)	6 (75.0%)	
	RML	1 (33.3%)	2 (66.7%)	
	RUL	7 (38.9%)	11 (61.1%)	
Differentiation	Well	4 (21.1%)	15 (78.9%)	*0.017*
	Moderate	11 (39.3%)	17 (60.7%)	
	Poor	3 (100%)	0 (0.00%)	
EGFR mutation	positive	8 (57.1%)	6 (42.9%)	0.052
	negative	10 (27.8%)	26 (72.2%)	
EFHD2 expression	positive	16 (61.5%)	10 (38.5%)	<*0.001*
	negative	2 (8.3%)	22 (91.7%)	

^a^*P*-value were performed by Chi-square or Fisher’s exact test.

### Mass spectrometric analysis

Membrane proteins were extracted using compartmental protein extraction kits CNM (BioChain Institute), which utilized chemicals to separate and purify cytoplasmic proteins, nuclear proteins, and membrane proteins individually. The membrane proteins were further separated by SDS-PAGE and divided into ten gel fractions. Each gel fragment was subjected to in-gel trypsin digestion, and then the tryptic peptides were injected into the linear ion trap-Fourier transform ion cyclotron resonance mass spectrometer (LTQ-FTICR MS) (Thermo Electron) for mass analysis. The survey scan (*m/z* range: 320–2,000) was performed in FTICR MS with a mass resolution of 100,000 at *m/z* 400. The top ten most abundant multiply charged ions were sequentially isolated for MS/MS assay by LTQ. MaxQuant^27^ and MaxLFQ^28^ softwares were used for protein identification and label-free quantitative analysis, respectively. The significance threshold for the identification was set as *P* < 0.01.

### Western blot analysis

Proteins were separated using 10% or 12.5% SDS-PAGE and transferred onto a PVDF membrane via electroblotting at 400 V at 4 °C for 3 hr in 25 mmol/L Tris-HCl, 197 mmol/L glycine, and 13.3% (v/v) methanol. Then, 5% (w/v) skim milk in TBST was used for the blocking reaction, and the primary antibodies were incubated at room temperature overnight. After TBST washes, horseradish peroxidase-conjugated secondary antibodies were further incubated at room temperature for 1 hr. Immunoreactive signals were revealed using an enhanced ECL substrate according to the manufacturer’s instructions (NEN Life Science). The primary antibodies used in this study included EFHD2 (ab106667; abcam), E-cadherin (#5296; Cell Signaling), vimentin (#3932; Cell Signaling), CAV1 (#3238; Cell Signaling), and β-actin (ab8226; abcam). The original images of Western blot assays are shown in Supplemental Fig. S1.

### Immunohistochemical assay

Immunohistochemical assays (IHCs) were performed using an automatic BenchMark XT staining machine (Ventana Medical Systems) iVIEW 3,3-diaminobenzidine (DAB) detection kit (Ventana Medical Systems). Paraffin sections (4 µm) containing human lung adenocarcinoma tissues were deparaffinized, hydrated, and heated to 95–100 °C for 4 min to induce antigen retrieval. After inactivating endogenous peroxidase activity, rabbit anti-human EFHD2 polyclonal antibody (#ab119119; abcam; 1:1,200) was used to perform IHC staining. Tissue sections were finally incubated with iVIEW copper for 4 min to enhance signal intensity. Then, samples were counterstained with hematoxylin, dehydrated, mounted, and observed using an Eclipse E600 light microscope (Nikon). All staining results were evaluated by an experienced histologist.

### Wound healing assay

The *in vitro* migration assay was performed using a Culture-Insert well (ibidi). Cancer cells (4.5 × 10^4^ cells) were cultured in suitable media in the device for 24 hr. After removal of the Culture-Insert, cancer cells were cultured for an additional 8 hr. The migration distance of cancer cells was recorded, and the migration area was measured using ImageJ software.

### Matrigel invasion assay

For *in vitro* invasion assay, cancer cells (1.5 × 10^5^ cells in 200 µL) were suspended in the upper half of a PET membrane transwell insert chamber (BD Biosciences), which was coated with Matrigel (1 mg/mL; BD Biosciences), on a 24-well plate. Medium supplemented with 10% FBS was added as a chemoattractant to the lower half. After incubation at 37 °C for 24 hr, cancer cells that passed through the insert were fixed with 3.7% formalin (Sigma-Aldrich) and stained with 0.1% crystal violet (Sigma-Aldrich).

### Quantitative polymerase chain reaction (qPCR)

Total RNA was extracted with TRIzol reagent (Invitrogen). RT-PCR was performed using 1 μg of sample and the MMLV First-Strand synthesis kit (GeneDireX), and a ten-fold dilution of the RT-PCR product was applied for qPCR analysis. qPCR was performed using KAPA SYBR® FAST qPCR Master Mix Kit (Kapa Biosystems) by the LightCycler 480 apparatus (Roche). GAPDH served as an endogenous control. Specific DNA expression was estimated by the comparative Ct method using 2^−ΔΔCt^. Primer information is provided in Supplementary Table 1.

### Statistical analysis

The data are displayed as the means ± SD and categorical data are presented as frequencies and proportions. The significance of differences was examined by Student’s *t*-test for continuous variables as well as Chi-square test or Fisher’s exact test for categorical variables as appropriate. Overall survival and disease-free survival were determined by the Kaplan-Meier method and compared with using the log-rank test. Multivariable analysis of the independent factors associated with disease-free survival was performed using the Cox proportional hazard model. The statistical analysis of the data was performed using IBM SPSS Statistics 22 (IBM Co.). *P* < 0.05 was considered statistically significant.

## Results

### EFHD2 is expressed in highly metastatic tumor cell lines

**Figure 1:**
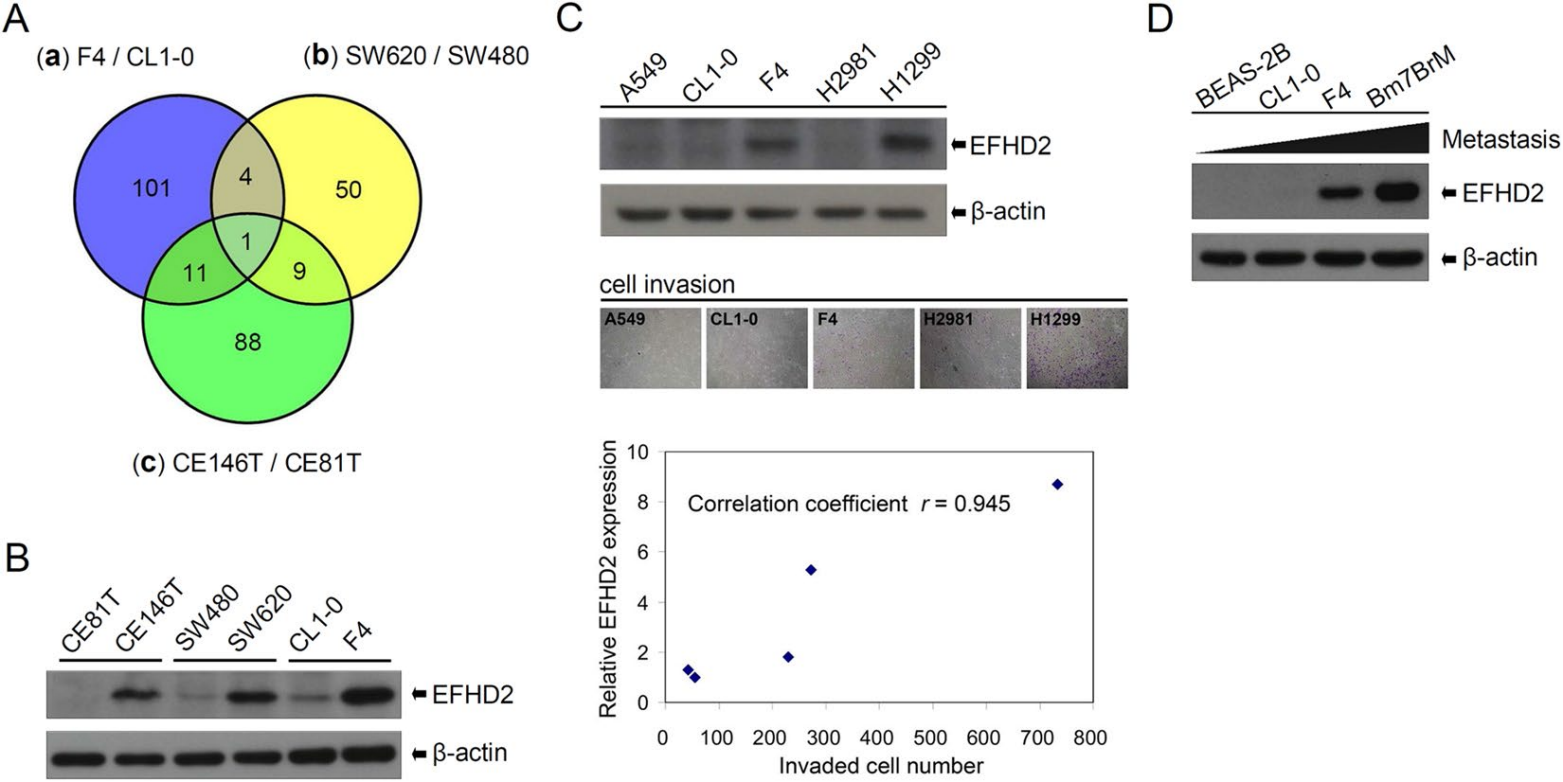
EFHD2 is expressed in highly metastatic tumor cells. (**A**) The intersection of membrane proteins with greater than 4-fold overexpression in highly metastatic tumor cell lines were analyzed using Venn diagram. (**B**) EFHD2 expression in the three pairs of tumor cells was determined by Western blot assay. (**C**) EFHD2 expression and the invasive ability of lung tumor cells were determined using Western blot and transwell assays, respectively. The linear correlation between the EFHD2 levels and the invasive ability was estimated by the Pearson correlation coefficient. EFHD2 levels of CL1-0 were used as the relative control and set as 1. (**D**) EFHD2 expression in BEAS-2B bronchial epithelial cells and lung tumor cells with different metastatic abilities (CL1-0, F4, and Bm7BrM) was determined by Western blot assay. β-actin, loading control.

To narrow down potential targets and test our assumption that common proteins are involved in metastasis in various tumor cells, we performed comparative proteomic analyses to identify metastasis-related markers. Three pairs of tumor cell lines with differential metastatic abilities were compared, including lung cancer cell lines CL1-0 and F4 (isogenic lines), colorectal cancer cell lines SW480 and SW620 (isogenic lines), and esophageal cancer cell lines CE81T and CE146T. The identified proteins with greater than 4-fold expression in highly metastatic tumor cell lines were further analyzed using a Venn diagram. The result revealed that only EF-hand domain-containing protein D2 (EFHD2) exhibited high expression in all highly metastatic tumor cell lines (Fig. 1A). Western blot validation revealed that EFHD2 was expressed at higher levels in tumor cells with more metastatic ability compared with those with less metastatic ability (Fig. 1B). To determine whether EFHD2 expression is correlated with the metastatic ability in lung tumor cells, we determined EFHD2 protein levels and invasive cell number using Western blot and transwell assays, respectively, in A549, CL1-0, F4, H2981, and H1299 cells. F4 and H1299 expressed increased endogenous levels of EFHD2 and exhibited an increased invasive ability compared with A549, CL1-0, and H2981. Accordingly, a positive correlation between EFHD2 protein levels and the invasive ability in lung tumor cells was noted, and the correlation coefficient was 0.945 (Fig. 1C). Moreover, the positive correlation was also observed in the isogenic lung tumor cell lines CL1-0, F4, and Bm7BrM (Fig. 1D). These results suggest that EFHD2 expression is associated with the increase of metastatic ability of lung tumor cells.

### EFHD2 is relevant to cancer recurrence in patients with stage I lung adenocarcinoma

**Figure 2:**
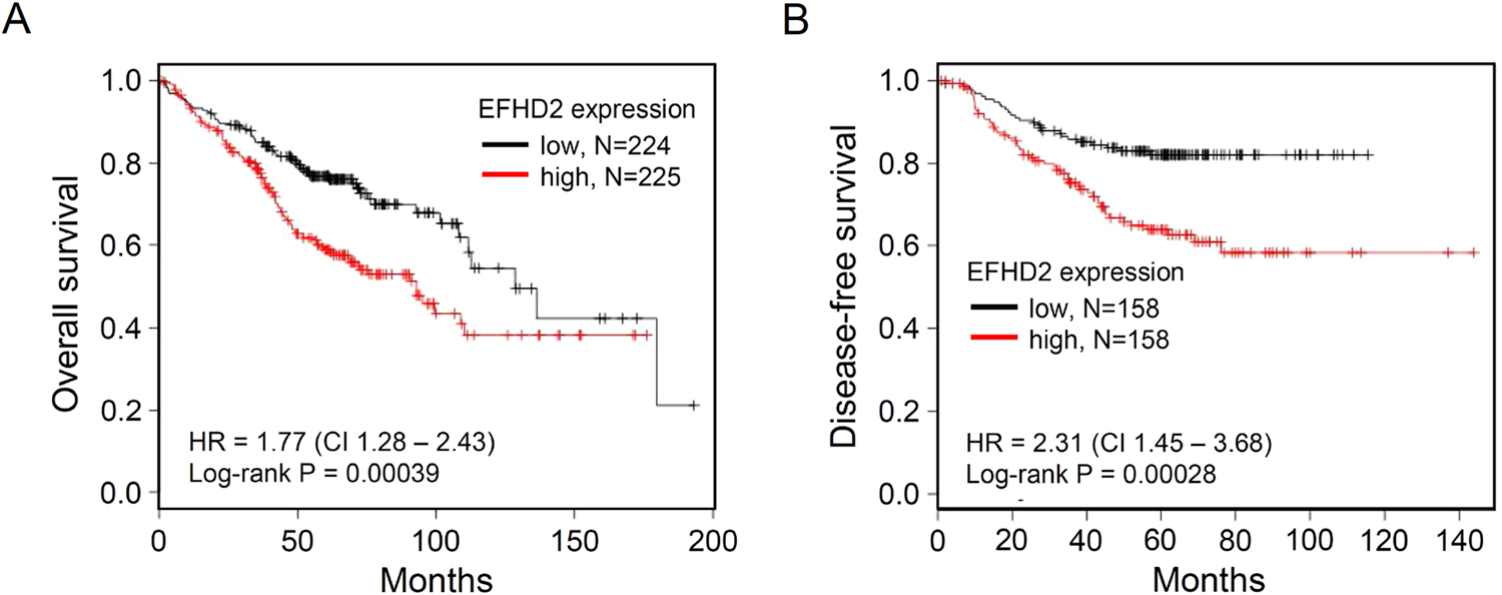
*In silico* analysis predicted that EFHD2 overexpression is correlated with poor survival in stage I lung adenocarcinoma patients. The correlation between EFHD2 mRNA levels and (**A**) overall survival or (**B**) disease-free survival of stage I lung adenocarcinoma patients was analyzed by Kaplan-Meier-plotter cancer database search.

**Figure 3:**
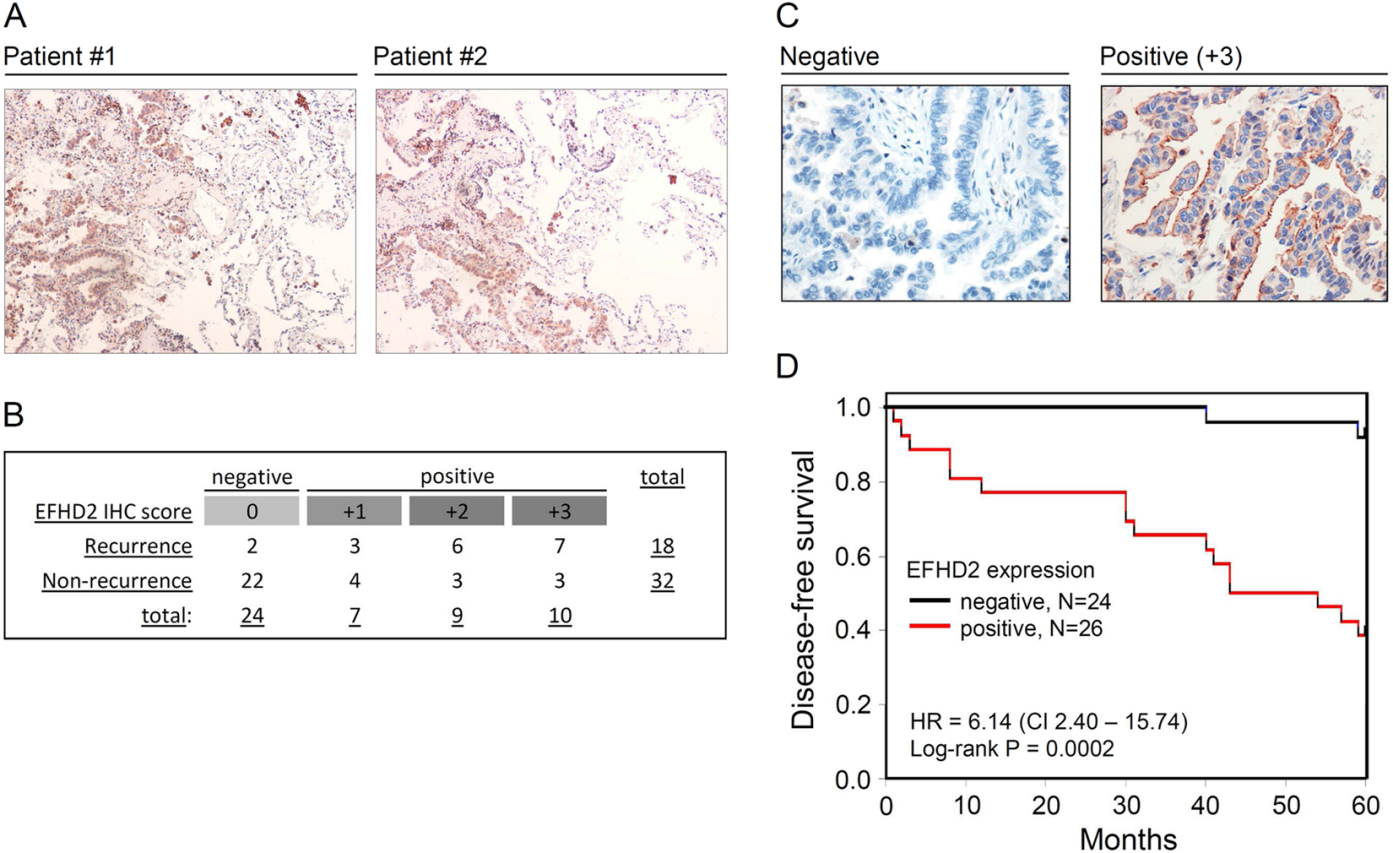
EFHD2 is associated with cancer recurrence in stage I lung adenocarcinoma patients. (**A**) IHC staining for EFHD2 expression in tumor tissue and normal tissue adjacent to lung adenocarcinoma from patients. Left part of tissue image shows tumor tissue, and right part shows tumor-adjacent normal lung tissue. Image magnification is 10X. (**B**) EFHD2 signal intensities were analyzed by IHC staining in 50 clinical tissue samples. The IHC signals were scored as 0, 1, 2, and 3, and a score ≧ + 1 indicated positive detection. (**C**) Representative photographs of EFHD2 signals in clinical samples. Image magnification is 400X. (D) Five-year disease-free survival calculated from Kaplan-Meier disease-free survival curve: EFHD2-negative vs. EFHD2-positive cases.

To evaluate EFHD2 expression in clinical samples, we performed IHC with an anti-EFHD2 antibody on the lung cancer tissue array BC041115a (US Biomax), which contains lung normal tissue, lung squamous cell carcinoma, and adenocarcinoma. EFHD2 was undetectable in lung normal tissue (Supplemental Fig. S2A). The percentages of cell expressing EFHD2 in cancer stage I, II, and IIIa were 36.8%, 37.5%, and 28.6%, respectively, in lung squamous cell carcinoma and 41.2%, 68.8%, and 50.0%, respectively, in lung adenocarcinoma (Supplemental Fig. S2B). This analysis revealed that EFHD2 expression did not correlate with cancer malignancy. Next, we determined whether EFHD2 expression was relevant to recurrence of early-stage NSCLC. The Kaplan-Meier-plotter cancer database search^29^ indicated that EFHD2 mRNA levels were significantly correlated with the overall survival and disease-free survival of stage I lung adenocarcinoma (Fig. 2A and B). Moreover, we performed a retrospective analysis of patients with stage I lung adenocarcinoma to determine the correlation between EFHD2 protein levels and postsurgical recurrence by IHC. Immunostaining revealed that EFHD2 was homogeneously expressed in cancer tissue, whereas negative signals were noted in normal tissue adjacent to lung cancer (Fig. 3A). In contrast to the non-recurrent group, the most recurrent group exhibited moderate to strong EFHD2 signals (Fig. 3B). It is especially noteworthy that lung adenocarcinoma without EFHD2 expression present dramatically lower recurrence (8.3%, 2/24) in comparison with EFHD2-positive lung adenocarcinoma (61.5%, 16/26) (Fig. 3B). The representative photographs of EFHD2 signals with negative and strong intensities are displayed in Fig. 3C. The disease-free survival was significantly worse in patient with positive EFHD2 signals (hazard ratio [HR], 6.14; 95% CI, 2.40–15.74) in Kaplan-Meier analysis (Fig. 3D). Log-rank tests revealed the disease-free survival rates were significantly different between patients who express EFHD2 and patients who do not express EFHD2 (*P* < 0.001) (Fig. 3D). In addition, the demographic, clinical and biochemical variables of patients were also evaluated for the consideration of recurrence, including age, gender, pathologic stage, tumor location, differentiation, smoking status, and EGFR mutation (Table 1). Univariate Cox regression analysis revealed that pathologic stage, differentiation, and EFHD2 expression were related to disease-free survival (Table 2). Multivariate Cox regression analysis revealed that only pathologic stage and EFHD2 expression were independent clinical predictors of disease-free survival of patients with stage I lung adenocarcinoma (*P* < 0.001) (Table 2). These data suggest that EFHD2 could serve as an independent marker to predict postsurgical recurrence of patients with stage I lung adenocarcinoma.

**Table 2:** Disease-free survival analysis of prognostic factors in stage I lung adenocarcinoma patients by Cox regression analysis.

Variables	Univariate analysis		Multivariate analysis	
	Hazard ratio (95% CI)	*P*-value	Hazard ratio (95% CI)	*P-* value
Age	1.023 (0.978–1.070)	0.314		
Gender *Male vs. female*	1.809 (0.713–4.595)	0.212		
Smoking status *Ever vs. never*	1.010 (0.391–2.607)	0.983		
Pathologic stage *1B vs. 1* *A*	3.576 (1.406–9.093)	*0.007*	8.158 (2.721–24.46)	<*0.001*
Tumor location *Lower lobe (LLL* + *RML* + *RLL) vs. upper lobe (LUL* + *RUL)*	0.812 (0.305–2.165)	0.678		
Differentiation *Poor vs. moderate*–*well*	9.240 (2.355–36.26)	*0.001*	2.501 (0.598–10.47)	0.209
EGFR mutation *Positive vs. negative*	2.135 (0.840–5.427)	0.111		
EFHD2 *Positive vs. negative*	11.05 (2.528–48.27)	*0.001*	21.55 (4.344–106.86)	<*0.001*

### EFHD2 promotes the metastatic abilities and epithelial-to-mesenchymal transition (EMT) of lung adenocarcinoma cells

**Figure 4:**
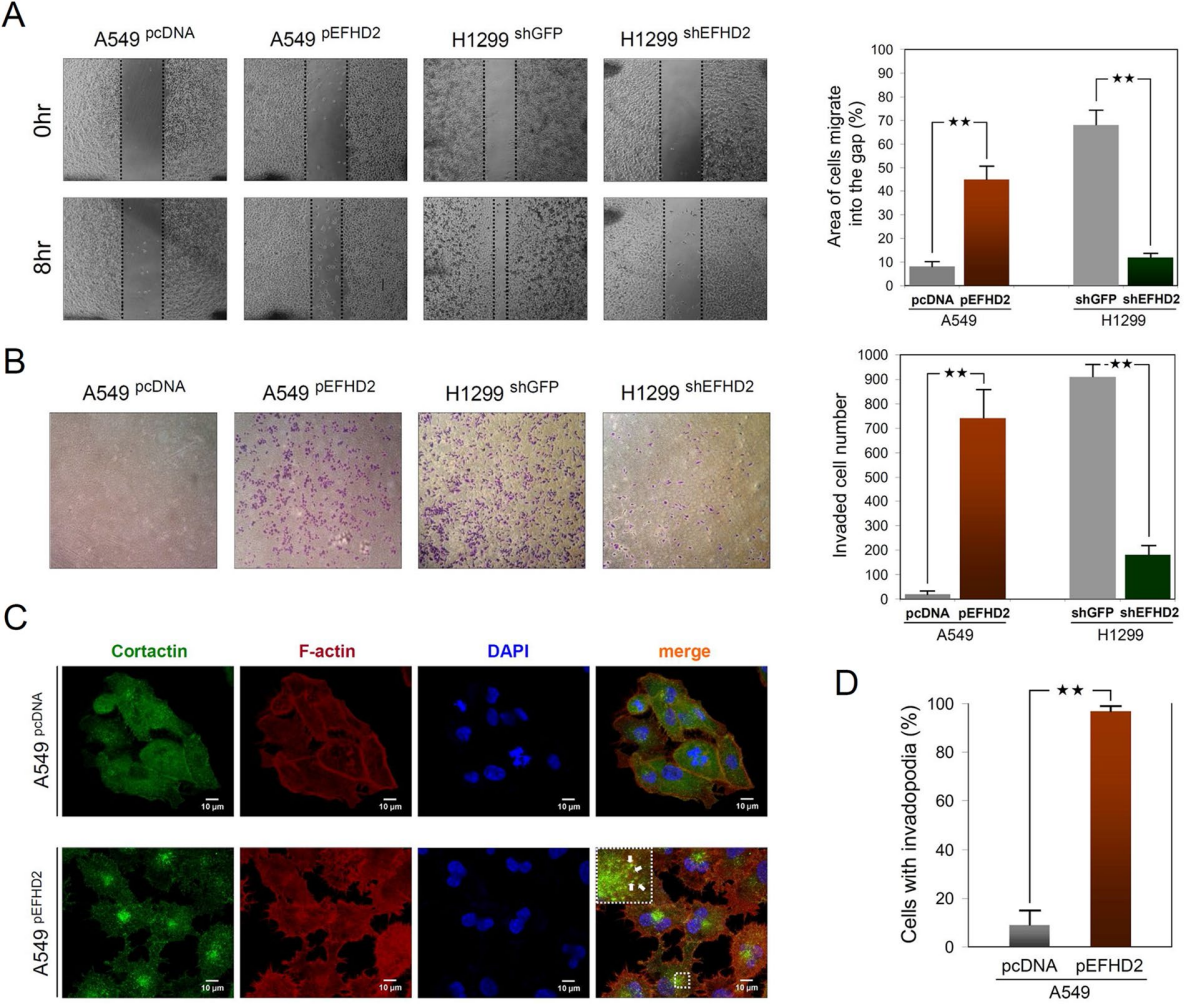
EFHD2 increases the metastatic abilities of lung adenocarcinoma cells. The effects of EFHD2 overexpression in A549 cells and EFHD2-knockdown in H1299 cells on metastatic abilities were determined. (**A**) Migration ability was analyzed by wound-healing assay. (**B**) Invasive ability was analyzed by transwell invasion assay. (**C**) Invadopodia were visualized by colocalized of cortactin (green) and F-actin (red). (**D**) Quantification of cancer cells with invadopodia. *N* = 100 cells/sample. **P < 0.01.

To determine whether EFHD2 contributes to the metastatic abilities of lung adenocarcinoma cells, we used the pcDNA vector to overexpress EFHD2 in A549 cells (low endogenous EFHD2 levels; Fig. 1C) and shRNA to knockdown EFHD2 in H1299 cells (high endogenous EFHD2 levels; Fig. 1C). EFHD2 overexpression and knockdown did not obviously affect cell growth based on MTT assays (Supplemental Fig. S3). EFHD2 overexpression significantly increased migration and invasiveness in A549, whereas EFHD2 knockdown had the opposite effects in H1299 (Fig. 4A and B). A previous study indicated that EFHD2 modulates actin bundling and F-actin structure and contributed to the formation of lamellipodia^13,14^, which represent an important driver during metastasis^30^. Accordingly, we examined the effect of EFHD2 on lamellipodia formation by confocal microscopy. The images demonstrated that EFHD2 increased invadopodia-like protrusive structures and the formation of invadopodia, which can be visualized by colocalization of cortactin and F-actin^31^ (Fig. 4C). In addition, EFHD2-overexpressing A549 cells exhibited significantly more cells with invadopodia structure compared with control cells (Fig. 4D).

**Figure 5:**
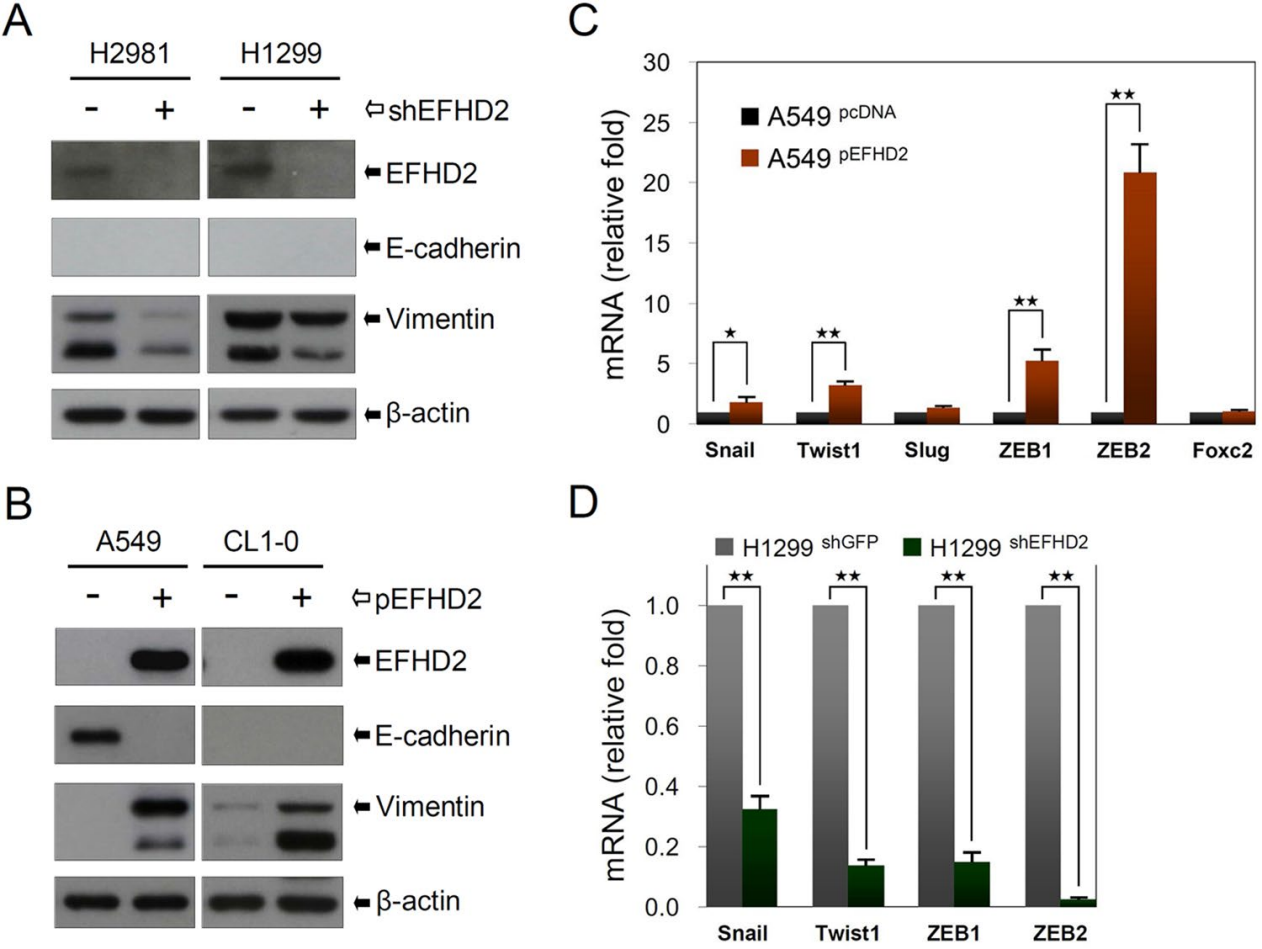
EFHD2 promotes the EMT. Western blot assay was used to determine the protein expression of EMT-related markers E-cadherin and vimentin in (**A**) EFHD2-knockeddown H1299 and H2981 cells and (**B**) EFHD2-overexpressing A549 and CL1-0 cells. β-actin, loading control. The qPCR assay was used to determine the mRNA expression of EMT-related transcriptional factors in (**C**) EFHD2-overexpressing A549 cells and (**D**) EFHD2-knockeddown H1299 cells. GAPDH, normalized control.

When tumor cells disseminate from the primary lesion to colonize distant organs, EMT is an important step during the initiation of metastasis^32^. To determine whether EFHD2 regulates the EMT program, we determined the expression of EMT-related proteins after EFHD2 overexpression or knockdown. Western blot assay indicated that EFHD2 knockdown decreased the expression of the mesenchymal cell marker vimentin in H1299 and H2981 cells (Fig. 5A). In contrast, EFHD2 overexpression increased the expression of the mesenchymal cell marker vimentin and reduced the expression of the epithelial cell marker E-cadherin in A549 and CL1-0 cells (E-cadherin is undetectable in CL1-0) (Fig. 5B). In addition, EFHD2 increased the expression levels of EMT-related transcriptional factors Snail, Twist1, ZEB1 and ZEB2 in A549 cells (Fig. 5C), but EFHD2 knockdown decreased the expression of these factors (Fig. 5D). Collectively, these results suggest that EFHD2 contributes to the promotion of EMT and metastasis in lung adenocarcinoma cells.

### EFHD2 promotes the EMT through inhibition of CAV1

**Figure 6:**
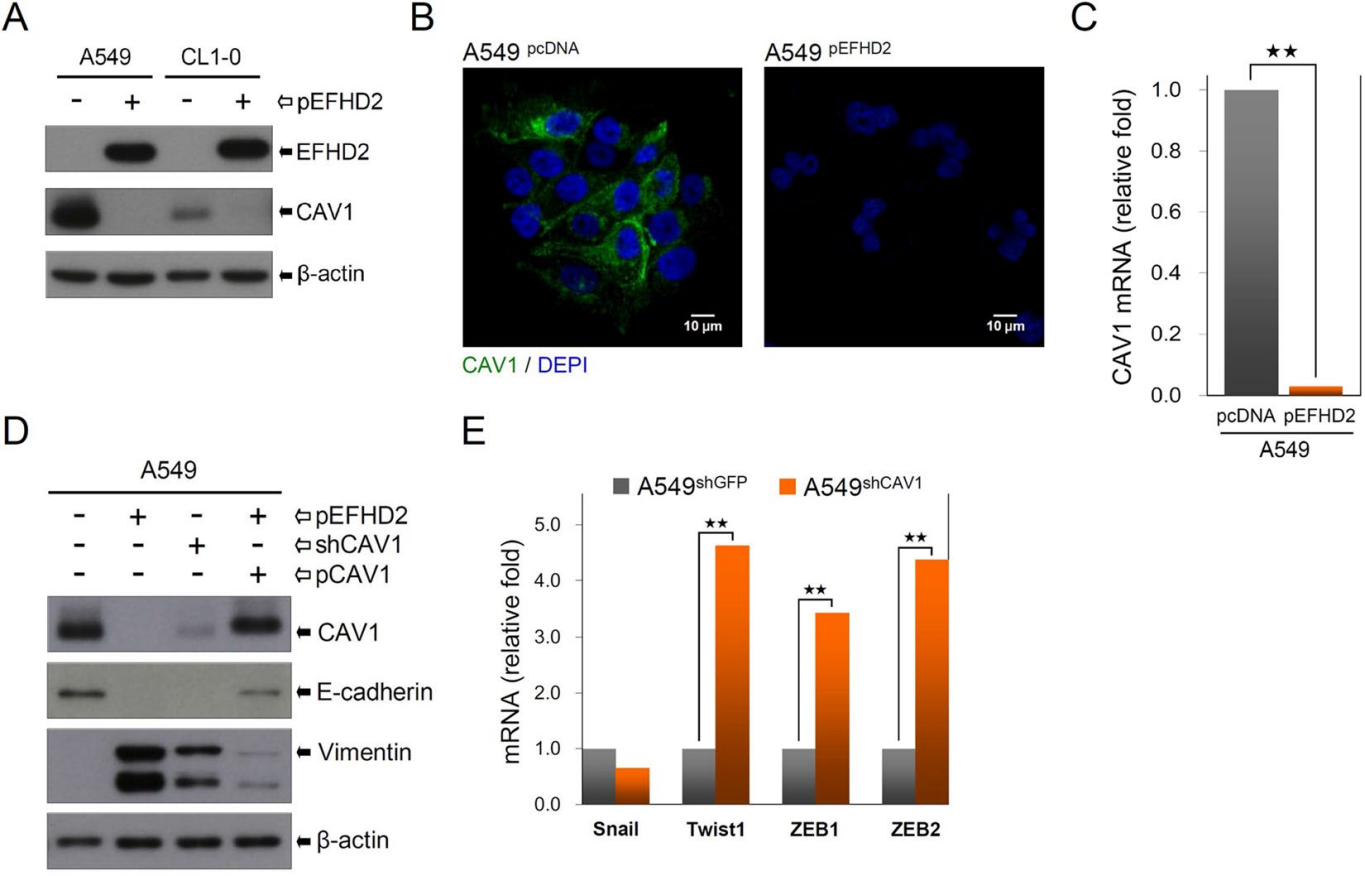
EFHD2 promotes the EMT partly through inhibition of CAV1. (**A**) The effect of EFHD2 on CAV1 expression in A549 and CL1-0 cells was determined by Western blot assays. β-actin, loading control. (**B**) CAV1 protein in EFHD2-overexpressing A549 cells and its control was analyzed by confocal microscopy. (**C**) CAV1 mRNA levels in EFHD2-overexpressing A549 cells and its control were analyzed by qPCR. (**D**) The effect of CAV1 knockdown in A549 cells and CAV1 re-expression in EFHD2-overexpressing A549 cells on E-cadherin and vimentin levels was determined by Western blot assay. (**E**) The effect of CAV1 knockdown on the expression of EMT-related transcriptional factors was determined by qPCR. ^★★^*P* < 0.01.

To investigate the underlying mechanism of EFHD2 in regulating EMT, a comparative proteomic analysis was performed to determine the protein signatures affected by EFHD2 overexpression. In total, 3904 proteins were identified in EFHD2-overexpressing and control A549, and protein quantification was performed using label-free proteomic methods^27,28^. Of these, 338 proteins exhibited greater than 5-fold overexpression, and 383 proteins exhibited less than 0.2-fold expression in EFHD2-overexpressing A549 cells compared with control cells (Supplementary Tables 2 and 3). Given that the function of EFHD2 is relevant to metastasis, we used a strategic combination search involving “the identified protein” and “metastasis” from the PubMed website to narrow down the candidates (Supplementary Fig. S4A). Certain candidates were validated by Western blot assay (Supplementary Fig. S4B). We noticed that caveolin-1 (CAV1) exhibited significantly decreased expression under EFHD2 overexpression conditions. EFHD2 dramatically suppressed CAV1 expression not only at protein levels in Western blot and confocal microscopy assays (Fig. 6A and B), but also at mRNA levels in qPCR analysis (Fig. 6C). To confirm whether EFHD2 regulates the EMT through inhibition of CAV1, we performed CAV1 knockdown in parental A549 and CAV1 rescue in EFHD2-overexpressing A549 cells. Direct CAV1 knockdown increased the expression of the mesenchymal cell marker vimentin and decreases the expression of the epithelial cell marker E-cadherin (Fig. 6D). In rescue experiments, the re-expression of CAV1 partly abolished EFHD2-induced EMT in A549 cells (Fig. 6D). In addition, CAV1 knockdown enhanced the expression of EMT-related transcriptional factors Twist1, ZEB1 and ZEB2 (Fig. 6E), which is similar to the effect of EFHD2 overexpression in A549 cells. Taken together, these results suggest that EFHD2 promotes the EMT through inhibition of CAV1.

## Discussion

Currently, several clinical and pathologic parameters have been proposed to distinguish those patients at increased risk of recurrence, including TNM classification, the standard uptake value in positron emission tomography scanning, histological differentiation, vessel invasion, lymphatic permeation, and pleural invasion^5,33^. These screening parameters may benefit patients with a low risk of postsurgical recurrence from avoiding unnecessary treatment, but whether these screening parameters improve the survival outcome of patients with a high risk of recurrence remains unknown. The use of molecular markers is an alternate method to define those patients with increased recurrence risk. Several molecular markers, such as gene mutation/deletion, DNA modification, miRNA, and alterations in protein or mRNA expression levels, are used to predict the recurrence potential in early-stage NSCLC. Of these, the gene signatures derived from comparative DNA microarray and qPCR analyses are the most widely proposed markers^34–36^. Due to poor reproducibility, gene markers have not applied to the clinic to date^37^. Several protein markers, including TS^38^, MACC1^39^, MIB-1 and Bcl-2^40^, and Ki-67 in combination with kRAS status^41^, predict postsurgical recurrence of stage I NSCLC. However, the predictive power of these markers exhibited unsatisfactory sensitivities and specificity, thus limiting their clinical application.

Recurrence of early-stage lung cancer could result from tumor cells disseminated during surgery or occult metastatic tumor cells that are undetected by standard diagnostic imaging^42^. The existence of occult metastatic tumor cells suggests that tumor cells could acquire metastatic characteristics and change their EMT feature at the primary site at a very early stage of disease. Consequently, it is possible to detect tumor cells with metastatic potential at the primary site in patients using a suitable method, such as IHC. Thus, precise prediction of recurrence at the early stage of cancer is theoretically feasible. Tissue is the most direct specimen for monitoring pathological changes in diseases, but the acquisition of tissue samples is not straightforward given the need for an invasive procedure. Surgical resection is the standard treatment for patients with early-stage lung cancer; thus, the measurement of diagnostic parameters from tissue specimens is practical. In this study, we utilized cancer database analysis and a cohort using an IHC approach to examine the correlation between EFHD2 and cancer recurrence. Our results revealed that EFHD2 was significantly correlated with postsurgical recurrence of patients with stage I lung adenocarcinoma (Fig. 3B; Table 1). EFHD2 measurements can detect recurrent stage I lung adenocarcinoma with a sensitivity of 88.9%, a specificity of 68.8% (p < 0.001, Chi-square test). Thus, EFHD2 could serve as a potential predictor for this disease.

EFHD2 was first identified in the immune system^7^. Currently, EFHD2 plays an important role in the nervous system^43^, and its functions are associated with several neuropathological disorders, including Parkinson’s disease and Alzheimer’s disease^10–12^. Until recently, EFHD2 was recognized to function in cancer metastasis. The study showed that EFHD2 overexpression induced pulmonary metastasis in highly invasive B16F10 melanoma cells, whereas its knockdown led to metastatic inhibition^15^. In the present study, we hypothesized that tumor cells use a similar mechanism to promote metastasis at a very early stage of disease and accordingly identified EFHD2 from highly metastatic tumor cell lines (Fig. 1). EFHD2 increased cell migration and invasion and promoted the EMT feature of lung adenocarcinoma cells (Figs 4 and 5), which is consistent with the previous finding^17^ and confirmed the critical role of EFHD2 in cancer metastasis. A growing body of evidence indicated that the actin-binding and F-actin bundling activities of EFHD2 were important for the regulation of cell spreading and migration through modulating actin-related membrane dynamics^13,14^. Actin-binding sites are located in the three major domains of EFHD2: polyA, EF-hand, and coiled-coil domain^13^. A recent structural study indicated that the F-actin bundling activity of EFHD2 depended on the structural rigidity of F-actin binding sites in the presence of Ca^2+^, and the C-terminal coiled-coil domain of EFHD2 is necessary for F-actin bundling^44^. Our confocal microscopy assay revealed that EFHD2 increased F-actin structure and the formation of lamellipodia (Fig. 4C and D), which is consistent with the observation that EFHD2 enhanced metastatic abilities in lung adenocarcinoma cells. The finding of EFHD2 was based on our originally risky hypothesis; thus, we assessed whether the hypothesis was applicable to other types of tumor cells. Based on our limited examination, we found that EFHD2 was expressed in numerous types of tumor cells with highly metastatic potential, including breast cancer, pancreatic cancer, and gastric cancer, but it is undetectable in liver tumor cells. These observations indicate that EFHD2 could contribute to the metastatic ability in a broad spectrum of cancers.

Past studies demonstrated that EFHD2 was localized to the membrane raft and the cytosol in mouse cells^9,44^. In human lung adenocarcinoma, IHC staining revealed that the strongest signals were located in the membrane, whereas weaker signals were noted in the cytosol (Fig. 3C). Whether EFHD2 translocates into the nucleus remains unknown due to a lack of experimental evidence. The primary sequence of EFHD2 does not possess a nuclear localization signal (NLS) or highly relevant sequence that is predicted to promote nuclear retention^45^. Consequently, although EFHD2 is capable of inducing the expression of multiple EMT-related transcriptional factors, such as Snail, Twist1, ZEB1 and ZEB2, and whether EFHD2 directly regulates these factors remains unknown.

CAV1 plays a negatively regulatory role in lung cancer development^20,21^. Our finding demonstrated a novel CAV1-related pathway in that EFHD2 enhanced metastatic abilities and promoted EMT partly through CAV1 inhibition (Fig. 6). EFHD2 increased the expression of early endosome antigen 1 (EEA1) in lung tumor cells (Supplementary Fig. S5) indicating that EFHD2 increases cellular endocytosis activity. This phenomenon is consistent with the previous finding that CAV1 knockdown increased endocytosis^22^, which is involved in the regulation of cell migration and cancer metastasis^23^. However, reduced CAV1 protein levels were observed in most tumor cells compared with adjacent normal cells (49/50 cases) in our IHC analysis. In addition to EFHD2, these results indicate that CAV1 could be regulated by multiple factors in pathological conditions. Given that the reduction of CAV1 was not significantly correlated with postsurgical recurrence of patients with stage I lung adenocarcinoma, CAV1 is unsuitable as a coordinated molecule to improve the predictive power of EFHD2.

Advances in diagnostic technology increase the opportunity for the early diagnosis of lung cancer (Supplemental Fig. S6), implying that the screening for early-stage disease patients with high risk of postsurgical recurrence will be required more than ever^46^. For recurrence prevention, the administration of adjuvant platinum-based chemotherapy is currently considered the standard of care of stage II and III patients with completely resected lung cancer^47^. However, whether adjuvant chemotherapy impacts patients with stages I lung cancer remains controversial^48^, and the debate may partly result from the lack of a precise method to identify patients with recurrence potential. In terms of personalized therapy, precise selection of patients is essential to avoid superfluous exposure to cytotoxic chemotherapeutic drugs and ensure the quality of life of patients. In the present study, we demonstrated that EFHD2 could serve as an independent marker to predict postsurgical recurrence of patients with stage I lung adenocarcinoma. However, a relatively small population sample recruited at a single institution indicates the limitation of marker applicability. A prospective study combined with the analysis of other suitable molecular predictors or pathological factors in a large number of patients will further refine the EFHD2 marker for clinical use.

## Electronic supplementary material

Supplementary Figures

Supplementary Tables
