# Publisher Correction: EFHD2 promotes epithelial-to-mesenchymal transition and correlates with postsurgical recurrence of stage I lung adenocarcinoma

**DOI:** 10.1038/s41598-018-19645-y

**Published:** 2018-01-18

**Authors:** Chi-Chen Fan, Wei-Chung Cheng, Yu-Chuen Huang, Yuh-Pyng Sher, Nia-Jhen Liou, Yu-Chuan Chien, Pei-Shan Lin, Pei-Syuan Lin, Chung-Hsuan Chen, Wei-Chao Chang

**Affiliations:** 1 Department of Superintendent Office, Mackay Memorial Hospital, Taipei, Taiwan; 2 Department of Medical Laboratory Science and Biotechnology, Yuanpei University, Hsinchu, Taiwan; 3 Research Center for Tumor Medical Science, China Medical University, Taichung, Taiwan; 4 Graduate Institute of Biomedical Sciences, China Medical University, Taichung, Taiwan; 5 School of Chinese Medicine, China Medical University, Taichung, Taiwan; 6 Genetics Center, Department of Medical Research, China Medical University Hospital, Taichung, Taiwan; 7 Center for Molecular Medicine, China Medical University Hospital, Taichung, Taiwan; 8 Genomics Research Center, Academia Sinica, Taipei, Taiwan; 9 Institute of Biochemistry & Molecular Biology, National Yang-Ming University, Taipei, Taiwan; 10 Department of Chemistry, National Taiwan University, Taipei, Taiwan; 11 Institute of Atomic & Molecular Sciences, Academia Sinica, Taipei, Taiwan

Correction to: *Scientific Reports* 10.1038/s41598-017-15186-y, published online 03 November 2017

The original HTML version of this Article contained a typographical error in the publication date ‘03 November 2017’ which was incorrectly given as ‘06 November 2017’. This has now been corrected in the HTML version of the Article.

