# Supplementary material for: EFHD2 promotes epithelial-to-mesenchymal transition and correlates with postsurgical recurrence of stage I lung adenocarcinoma
Source: Sci Rep. 2017 Nov 3;7:14617. doi: 10.1038/s41598-017-15186-y (PMC5668280; doi:10.1038/s41598-017-15186-y)
Supplement: Supplementary file 1 — Supplementary Figures [file 41598_2017_15186_MOESM1_ESM.pdf]

Supplementary information

EFHD2 promotes epithelial-to-mesenchymal transition and correlates with postsurgical recurrence of stage I lung adenocarcinoma

Chi-Chen Fan, Wei-Chung Cheng, Yu-Chuen Huang, Yuh-Pyng Sher, Nia-Jhen Liou, Yu-Chuan Chien, Pei-Shan Lin, Pei-Syuan Lin, Chung-Hsuan Chen, Wei-Chao Chang.

Supplementary Figure S1

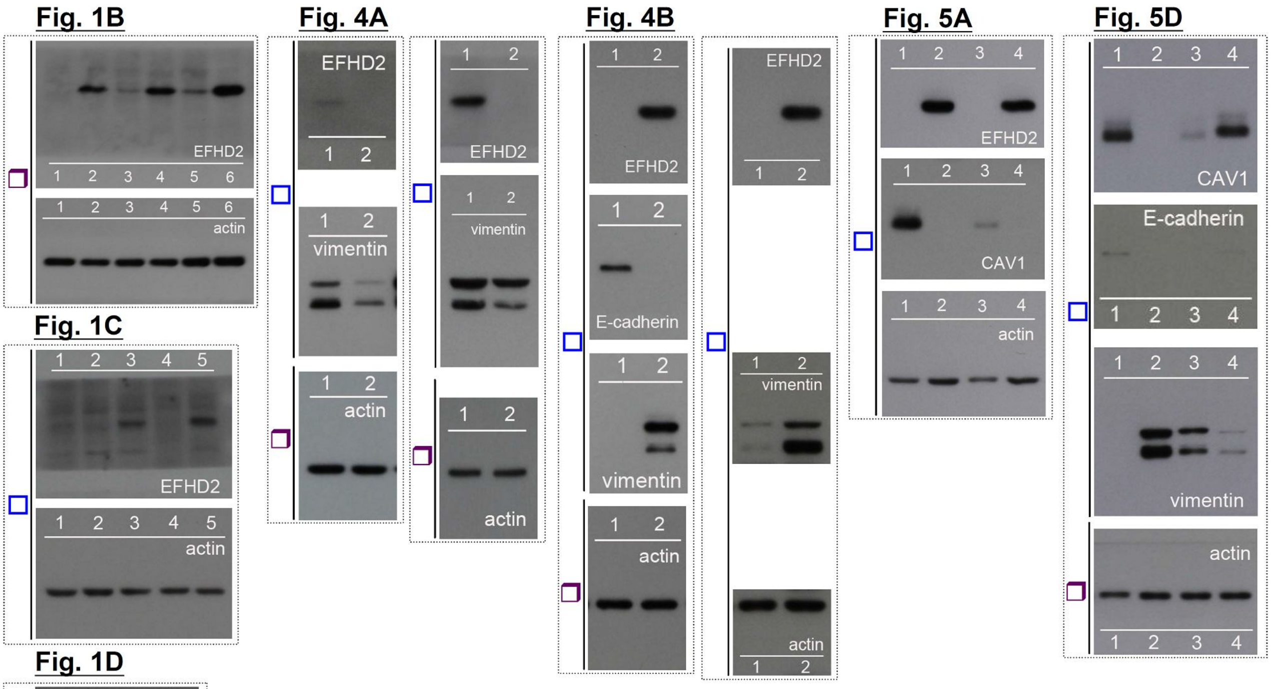

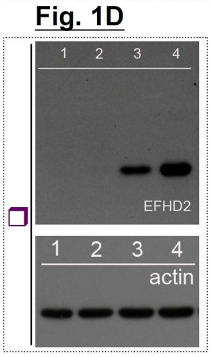

Supplementary Figure S1. The original images of western blot assays.

We use the following symbols to show the grouping of blots coming from:

□: cropped from different parts of the same gel, or

❐: from different gels of the same samples

Supplementary Figure S2

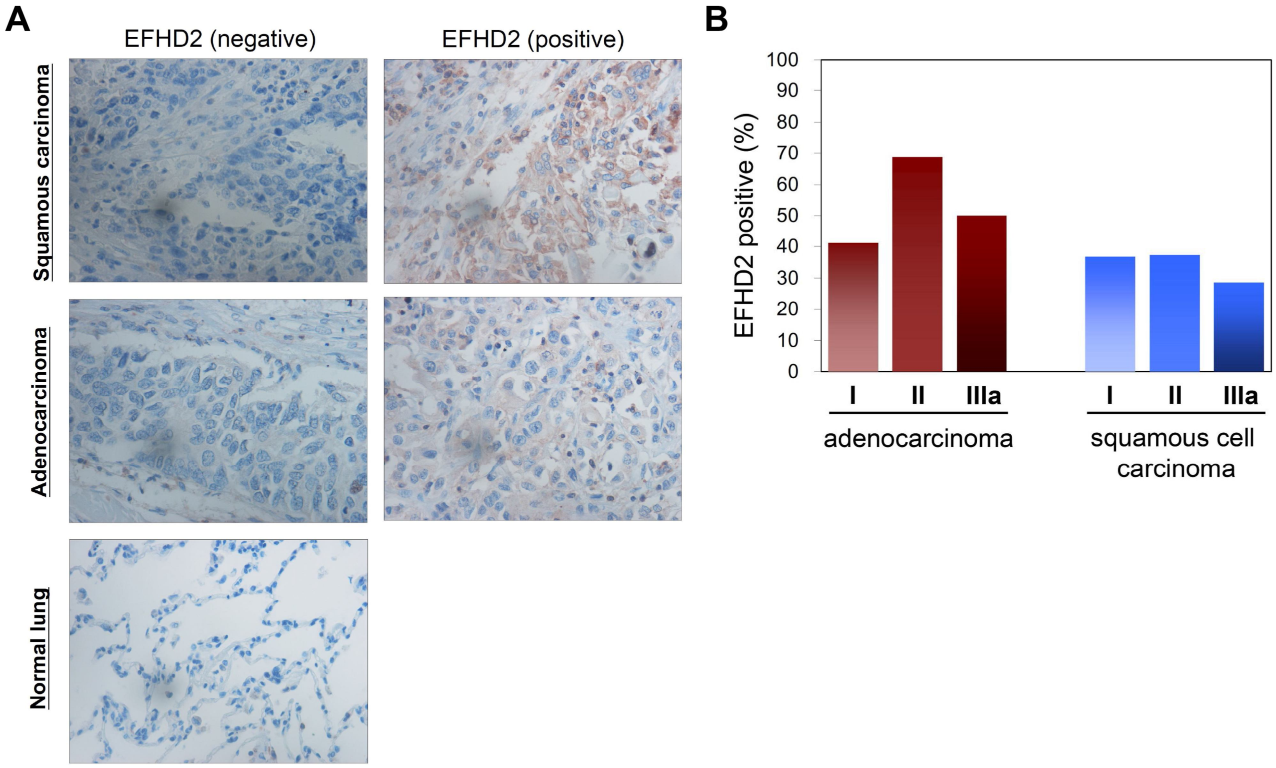

Supplementary Figure S2. EFHD2 expresses in lung tumor tissues. EFHD2 expression in clinical tissues was determined on the lung cancer tissue array BC041115a (US Biomax) using IHC. (A) Representative photographs of EFHD2 signals in lung squamous cell carcinoma, adenocarcinoma, and normal tissues. Image magnification is 400X. (B) The percentages of cell expressing EFHD2 in various stages of lung squamous cell carcinoma and adenocarcinoma were plotted.

Supplementary Figure S3

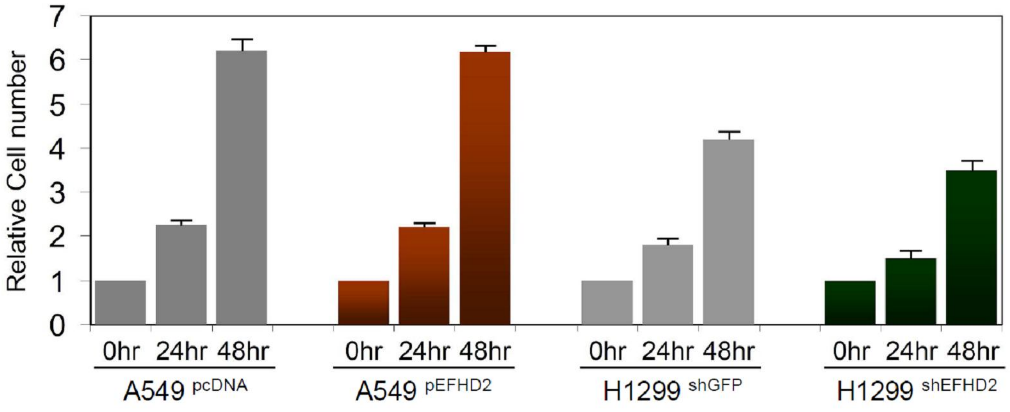

Supplementary Figure S3. EFHD2 overexpression and knockdown did not significantly affect the growth of lung adenocarcinoma cells. Cell growth of EFHD2-overexpressing A549 cells and EFHD2-knockeddown H1299 cells was compared with their control cells. The relative cell number was determined using MTT assays after 24hr and 48hr growth.

Supplementary Figure S4

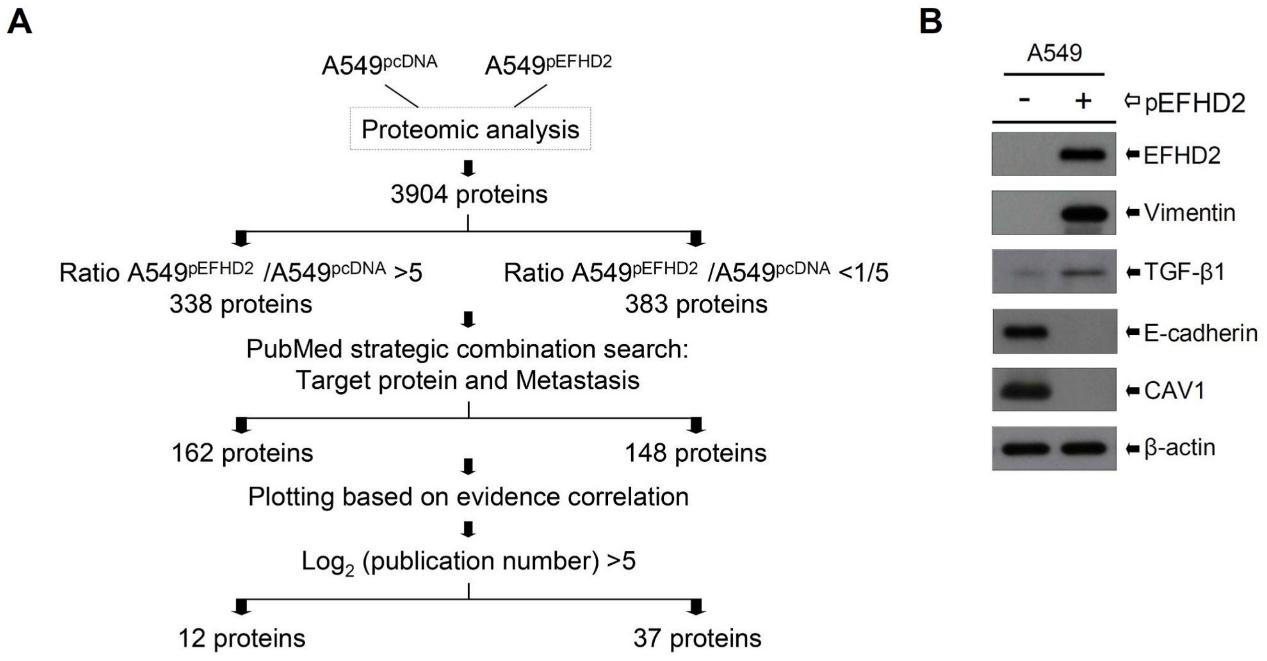

Supplementary Figure S4. The effect of EFHD2 on the expression of metastasis-related proteins in A549 cells. (A) Flowchart for the identification of metastasis-related proteins that were affected by EFHD2 overexpression. Data of comparative proteomics were divided into two parts: up-expressed proteins with greater than 5-fold expression and down-expressed proteins with lower than 0.2-fold expression in EFHD2-overexpressing A549 cells compared with control cells. The candidates were further analyzed based on a strategic search via the combination of "the identified protein" and "metastasis" from the PubMed website. Proteins with high evidence correlation (>32 publication number) were selected. (B) Certain candidates were validated by Western blot assays. β-actin, loading control.

Supplementary Figure S5

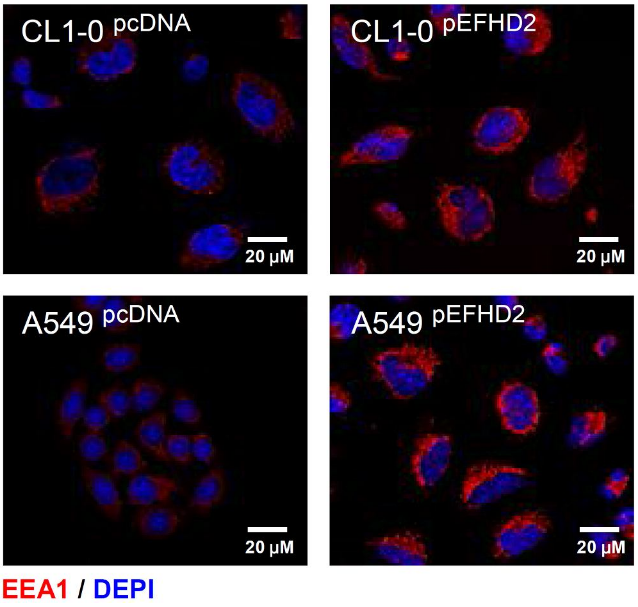

Supplementary Figure S5. EFHD2 increases cellular EEA1 levels. EEA1 protein levels in EFHD2-overexpressing CL1-0 and A549 cells were compared with their control cells by confocal microscopy. The nuclei were stained with DEPI (blue).

Supplementary Figure S6

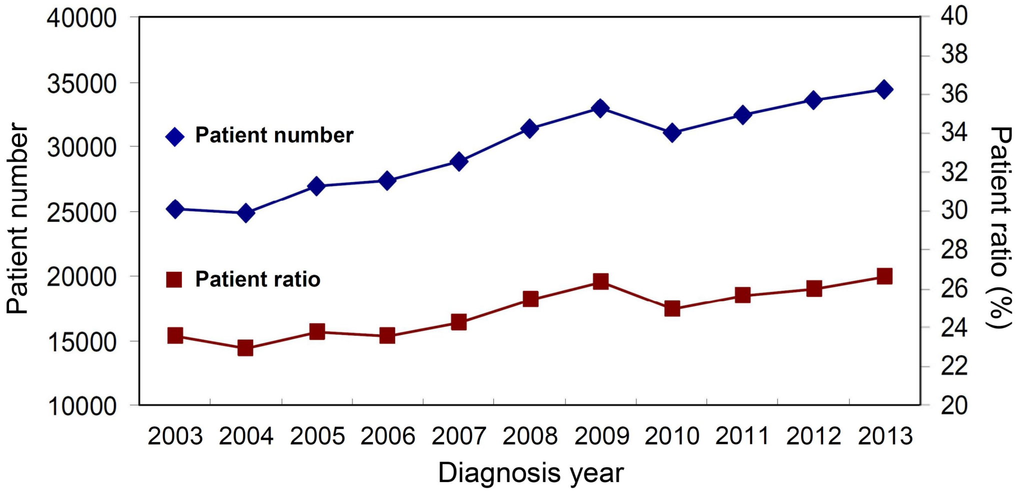

Supplementary Figure S6. Patient number of lung cancer and patient ratio of stage I disease who diagnosed in the United States. The graph showed that stage I lung cancer patients diagnosed in 2003 to 2013 from 1595 hospitals cancer cases reported to the National Cancer Database (NCDB) by tumor type and AJCC stage in the United States.
